# Establishment of a Triple Quadrupole HPLC-MS Quantitation Method for Dystrophin Protein in Mouse and Human Skeletal Muscle

**DOI:** 10.3390/ijms25010303

**Published:** 2023-12-25

**Authors:** Tsukasa Tominari, Masaru Takatoya, Toshiya Matsubara, Michio Matsunobe, Daichi Arai, Chiho Matsumoto, Michiko Hirata, Shosei Yoshinouchi, Chisato Miyaura, Yoshifumi Itoh, Hirofumi Komaki, Shin’ichi Takeda, Yoshitsugu Aoki, Masaki Inada

**Affiliations:** 1 Department of Biotechnology and Life Science, Tokyo University of Agriculture and Technology, Koganei, Tokyo 184-8588, Japan; 2 Life Science Research Center, Shimadzu Corporation, Nakagyo, Kyoto 604-8511, Japan; 3 Cooperative Major of Advanced Health Science, Tokyo University of Agriculture and Technology, Koganei, Tokyo 184-8588, Japan; 4 Inada Research Unit, Institute of Global Innovation Research, Tokyo University of Agriculture and Technology, Koganei, Tokyo 184-8588, Japan; 5 Kennedy Institute of Rheumatology, Nuffield Department of Orthopaedics, Rheumatology and Musculoskeletal Sciences, University of Oxford, Oxford OX3 7FY, UK; 6 Translational Medical Center, National Institute of Neuroscience, National Center of Neurology and Psychiatry, Kodaira, Tokyo 187-8551, Japan; 7 Department of Molecular Therapy, National Institute of Neuroscience, National Center of Neurology and Psychiatry, Kodaira, Tokyo 187-8502, Japan

**Keywords:** dystrophin, Duchenne muscular dystrophy, LC-MS

## Abstract

Duchenne muscular dystrophy (DMD) is the most common type of neuromuscular disease caused by mutations in the *DMD* gene encoding dystrophin protein. To quantitively assess human dystrophin protein in muscle biopsy samples, it is imperative to consistently detect as low as 0.003% of the dystrophin protein relative to the total muscle protein content. The quantitation of dystrophin protein has traditionally been conducted using semiquantitative immunoblotting or immunohistochemistry; however, there is a growing need to establish a more precise quantitative method by employing liquid chromatography-mass spectrometry (LC-MS) to measure dystrophin protein. In this study, a novel quantification method was established using a mouse experiment platform applied to the clinical quantification of human dystrophin protein. The method using a spike-in approach with a triple quadrupole LC-MS quantitated the amount of dystrophin in wild-type and human DMD transgenic mice but not in DMD-null mice. In conclusion, we established a quantitating method of dystrophin using HPLC-LC-MS with a novel spike-in approach. These results indicate that our methodology could be applied to several LC-MS devices to enable the accurate measurement of dystrophin protein in patients with DMD.

## 1. Introduction

Muscular dystrophy is characterized by weakness in and degeneration of skeletal muscle. Approximately 1 in 5000 male infants are diagnosed with Duchenne muscular dystrophy (DMD), which is an X-linked recessive muscular disorder. DMD is caused by disruption of the reading frame with mutations of the *DMD* gene, which encodes dystrophin, leading to the lack of functional dystrophin protein [1,2]. Dystrophin is an essential cytoskeletal protein that supports cell membranes and stabilizes myofiber structures by linking the intracellular actin filaments and extracellular matrix during the contraction and stretching of myofibers [3]. A lack of dystrophin causes muscle cell membrane disruption during the movements of myofibers, which leads to muscular inflammation and necrosis, resulting in fibrosis and fat accumulation in muscle tissues [4,5]. Becker muscular dystrophy (BMD) is a mild degenerative disease caused by mutations of the *DMD* gene, in which the restored open reading frame produces truncated partially functional dystrophin protein [2,3].

Recently, various therapeutic approaches have been developed for the treatment of DMD [6] including cell transplantation [7], read-through of stop codons [8], gene-addition with micro-dystrophin using adeno-associated virus (AAV) vector [9,10], and exon-skipping [11]. Anti-inflammatory approaches are also used for the treatment of DMD, including steroid therapy [12,13] and a novel inhibitor of prostaglandin D synthase, which has been evaluated in phase II clinical trials [14,15]. Exon skipping by antisense oligonucleotides (ASOs) is one of the most important therapies for DMD. ASOs restore the open reading frame through exon skipping, which converts out-of-frame deletions to in-frame deletions. Therefore, partially functional and truncated dystrophin protein is produced, leading to the improvement of muscle function, similar to BMD [16,17,18]. In 2020, a novel exon 53 skipping drug, NS-065/NCNP-01 (viltolarsen), received approval in Japan and from the FDA for the treatment of DMD [19,20]. Since exon-skipping therapies for DMD treatment partially recover the dystrophin protein, the establishment of a quantitative method is an important issue. It is difficult to accurately quantify dystrophin protein by mass spectrometry (MS) because dystrophin protein only accounts for 0.002–0.03% of total muscle protein [1]. Therefore, semiquantitative methods, including immunoblotting and immunostaining of tissue sections, have been predominantly used to assess the drug efficacy of DMD treatment [21]. Reverse transcriptase-polymerase chain reaction (RT-PCR) of dystrophin mRNA is also widely used to elucidate the dystrophin restoration by exon skipping; however, the mRNA expression does not always reflect the absolute amount of protein [21,22].

With regard to the MS-based quantification of dystrophin in human muscle biopsy specimens, one study reported the methodology with human endogenous dystrophin peptides measured with stable isotope-labeled mice dystrophin peptides. Human patient-derived samples mixed with SILAC (stable isotope [SI] labeling by amino acids in cell culture)-labeled mice-derived muscle samples (as the internal standard) were measured by nano liquid chromatography Orbitrap mass spectrometry (nanoLC-Orbitrap MS [23]. Another study reported the development of a method for the absolute quantitation of dystrophin protein using SILAC-labeled human myotubes with ultra-performance LC (UPLC)-Orbitrap MS [24]. It is well-known the LC-MS device is frequently used in clinics and/or hospitals to measure various analytical samples from patients; however, there is no established methodology for the quantification of dystrophin using a system with a triple quadrupole-based LC-MS device.

In this study, we developed a system using custom synthetic peptides with sequences identical to mouse and human dystrophin peptides as an internal control and established a method that enables the accurate quantitation of dystrophin protein by a spike-in approach using a system with a triple quadrupole HPLC-MS device. In this approach, isotope-labeled heavy peptides at known concentrations are simultaneously analyzed by LC-MS with sequence-matched endogenous peptides at unknown concentrations, and the concentration can be quantitated from the peak area ratio of the labeled heavy peptide to the endogenous peptide. Dystrophin protein derived from the mouse skeletal muscles of wild-type, DMD-null, human intact DMD sequence transgenic (hDMD-tg) mice, and human skeletal muscle biopsies were quantified. With the establishment of the method, several types of LC-MS devices allow the reproducible measurement of dystrophin protein for the therapeutic evaluation and clinical diagnosis of DMD patients.

## 2. Results

### 2.1. Optimization of LC-MS Conditions Using the Analysis of Dystrophin Peptides

**Figure 1:**
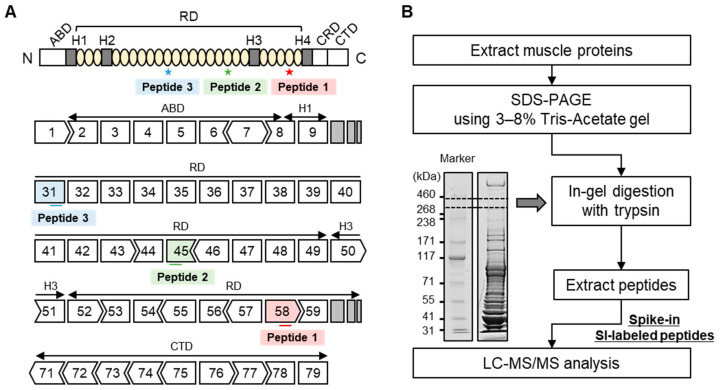
The location of three custom peptides on mouse dystrophin and flowchart of the SI-labeled peptides spike-in approach. (**A**) The location of three custom peptides on the protein structure (upper) and exon sequences (lower) of full-length mouse dystrophin. (**B**) Mouse TA muscles or human muscle biopsy samples were homogenized to extract muscle proteins. Total muscle protein was loaded onto 3–8% Tris-Acetate gel and separated by SDS-PAGE. The gel area between approximately 300 and 450 kDa, which contained dystrophin protein, was excised and in-gel tryptic digestion was performed. SI-labeled peptides were added to extracted samples and analyzed by LC-MS/MS.

**Figure 2:**
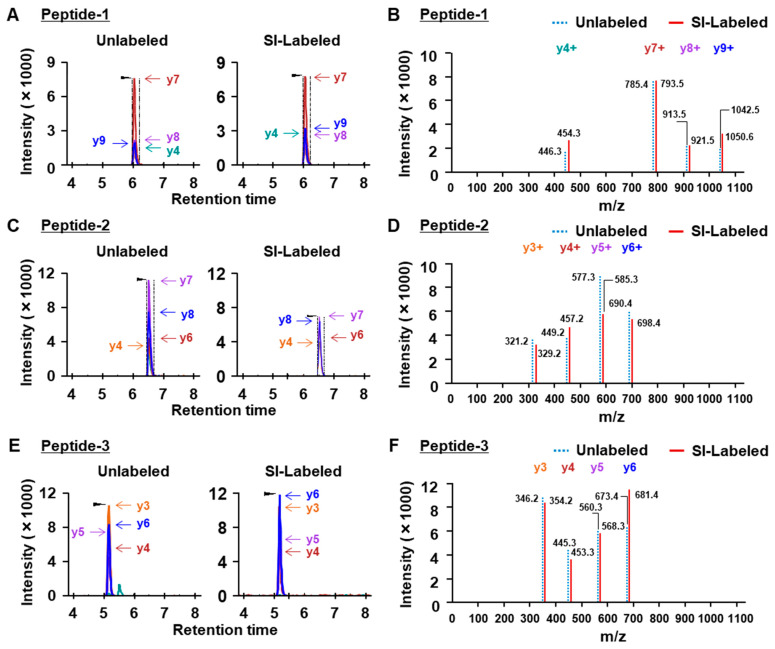
MRM chromatograms of three custom synthetic peptides with and without SI labeling. The three unlabeled (10 nM) or SI-labeled peptides (10 nM), IFLTEQPLEGLEK (peptide 1) (**A**,**B**), TLNATGEEIIQQSSK (peptide 2) (**C**,**D**), and VLSQIDVAQK (peptide 3) (**E**,**F**) were analyzed by LC-MS. The y-ion fragments of peptide-1 (**A**), peptide-2 (**C**), and peptide-3 (**E**) were detected at the same time point using the Skyline software program. The MS/MS spectra were shown at the retention time described in (**B**,**D**,**F**). Each MS/MS spectrum of the endogenous peptide exhibited −8 *m*/*z* relative to the SI-labeled peptide in the LabSolutions software program.

Dystrophin protein is thought to represent 0.002–0.03% of normal muscle protein, which is why it is challenging to use mass spectrometry for the quantitation of dystrophin protein. Recent reports have demonstrated the quantitation of dystrophin in human skeletal muscle using high-precision orbitrap mass spectrometry connected to a nano-HPLC or UPLC (ultra-performance LC) system [23,24]. In this study, we attempted to analyze dystrophin protein using triple quadrupole HPLC-MS. First, we analyzed three custom synthetic peptides with sequences identical to mouse and human dystrophin peptides (Figure 1A and Table 1). We prepared the peptide mixture of unlabeled and SI-labeled synthetic peptides at a concentration of 10 nM each to determine the retention time and LC-MS condition (Figure 1B). The peptide mixture was analyzed by LC-MS, and the analytical data were exported to a quantitative proteomics software program (Skyline, version 21.1) to select fragment ions exhibiting a strong peak. We determined the retention time (RT) and four fragment ions exhibiting higher ionization in each peptide for the quantitation of dystrophin by an LC-MS analysis (Table 2). Next, to increase the sensitivity for the ionization of fragment ions in each dystrophin peptide, we optimized the LC-MS method, including the Q1 pre-bias, CE, and Q3 pre-bias, using an LC-MS analysis software program (LabSolutions, version 5.113). As a result of the analysis of unlabeled and SI-labeled peptides using the optimized method, unlabeled and SI-labeled peptides showed a similar intensity on MRM chromatograms (Figure 2A,C,E), and each MS/MS spectrum of the unlabeled peptide showed −4 *m*/*z* (divalent ion) or −8 *m*/*z* (monovalent ion) in comparison to the SI-labeled peptide (Figure 2B,D,F). Finally, we optimized the LC-MS method based on these analyses and further analyzed the protein quantitation of dystrophin from mouse and human skeletal muscle.

### 2.2. Protein Quantitation of Dystrophin in Mouse Skeletal Muscle

**Figure 3:**
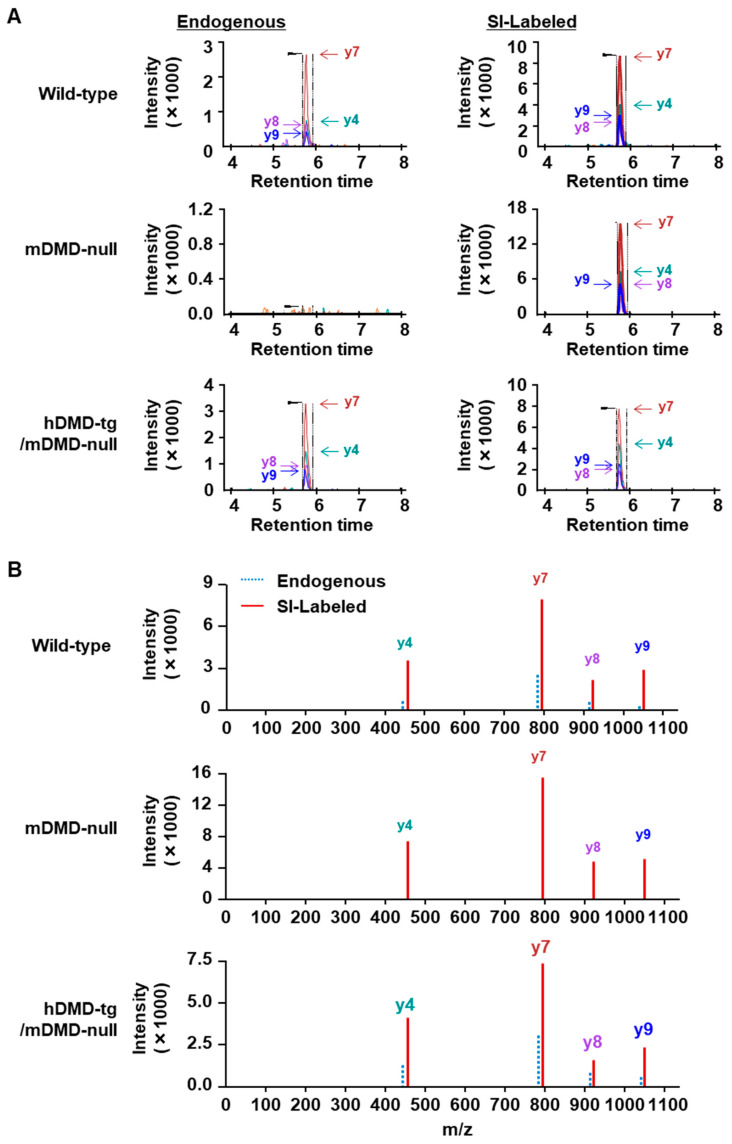
The analysis of dystrophin protein in mouse skeletal muscle by LC-MS. Endogenous peptides, a tryptic digested samples from 0.8 mg of total protein of wild-type, mDMD-null and hDMD-tg/mDMD-null mice, and SI-labeled peptides (10 nM), IFLTEQPLEGLEK (peptide 1), were analyzed by LC-MS. The MRM chromatogram (**A**) and MS/MS spectrum (**B**) are shown.

**Figure 4:**
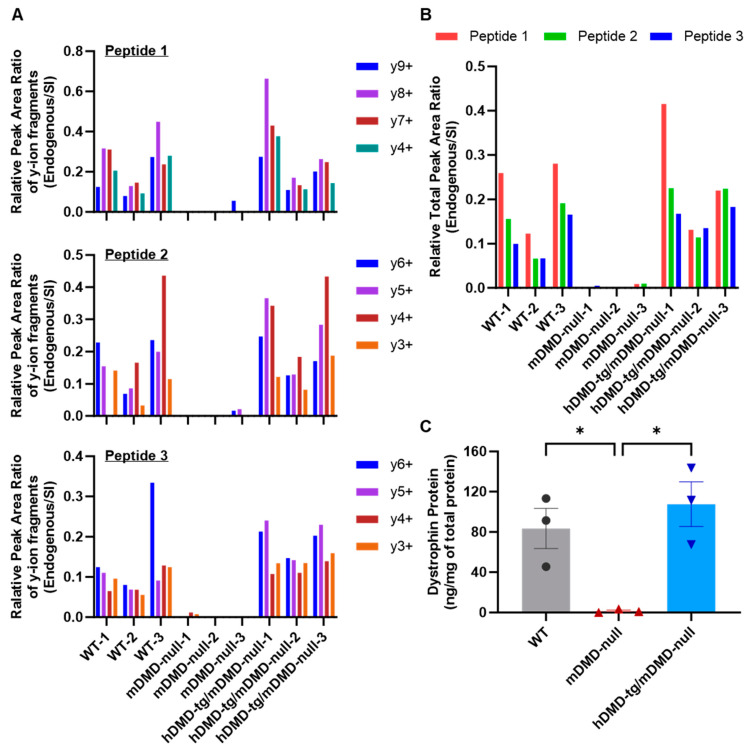
Accurate quantitation of dystrophin protein in mouse skeletal muscles. (**A**) The relative peak area ratio (endogenous/SI) of each MRM transition in three dystrophin peptides in mouse TA muscles. (**B**) The relative total area ratio (endogenous/SI) of three dystrophin peptides in mouse TA muscles. (**C**) The amounts of dystrophin protein from mDMD-null and hDMD-tg/mDMD-null mice were quantitated by LC-MS using the three SI-labeled peptides spike-in approach. The data are expressed as the mean ± SE of three mice with the individual datum point (black circle: each WT mouse, red triangle: each mDMD-null mouse, blue inverted triangle: each hDMD-tg/mDMD-null mouse). Asterisks indicate a significant difference between the two groups: * p < 0.05 by one-way ANOVA followed by Tukey’s test.

We analyzed total muscle protein isolated from TA in wild-type, mouse dystrophin-deficient (mDMD-null) mice, and transgenic mice expressing human dystrophin but not mouse dystrophin (hDMD-tg/mDMD-null mice). The mDMD-null mice were produced by the complete deletion of the 2.4 Mb *DMD* gene using a Cre-*loxP* system, which exhibits typical DMD symptoms, including muscle degeneration and regeneration [25]. The hDMD-tg/mDMD-null mice were produced by crossing hDMD-tg [Tg(DMD)72Thoen/J] mice, which were generated by ‘t Hoen et al. [26], with mDMD-null mice [27]. The 0.8 mg of total muscle protein from TA in each mouse was extracted and separated by SDS-PAGE with 3–8% Tris-Acetate gel, followed by in-gel tryptic digestion of excised gel containing an area of approximately 300–450 kDa. As an internal standard, SI peptides were added to tryptic digestion samples, and the amount of dystrophin protein was analyzed by LC-MS (Figure 1A). As a result, no endogenous signal was detected in mDMD-null mice, while the endogenous signal was detected in wild-type and hDMD-tg/mDMD-null mice (Figure 3A,B). The relative peak area ratio (endogenous/SI) of the LC chromatogram in each MS/MS transition (Figure 4A) and each peptide (Figure 4B) is shown. The endogenous peak areas could not be measured in mDMD-null mice. In mDMD-null mice, the y9-ion ([EQPLEGLEK]^+^) of peptide 1, y6 and y5-ions ([IQQSSK]^+^ and [QQSSK]^+^) of peptide 2, and y4 and y3-ion ([VAQK]^+^) and [AQK]^+^) of peptide 3 were slightly measured due to the detection of low signal intensity (Figure 4A); however, these signals might have been background noise since no MS/MS spectra were detected, as shown in Figure 3B. The amounts of endogenous dystrophin were determined from the average of the respective dystrophin amounts calculated by the relative total area ratio (endogenous/SI) of three dystrophin peptides. As shown in Figure 4C, the quantitative amounts of dystrophin protein were 83.43 ng ± 20.01 (mean ± SE) per mg of total muscle protein in wild-type mouse TA muscles, compared to 1.47 ng ± 1.01 (mean ± SE) per mg of total muscle protein in three mDMD-null mouse TA muscles. In three hDMD-tg/mDMD-null mouse TA muscles, the quantitated amount of dystrophin protein was 107.60 ng ± 22.07 (mean ± SE) per mg of total muscle protein. Additionally, we analyzed the mixed muscle lysates from hDMD-tg/mDMD-null mouse and mDMD-null mouse with 0%, 25%, 50%, 75%, and 100% dystrophin protein, and we detected at least 25% dystrophin protein of hDMD-tg/mDMD-null muscle protein (Supplementary Figure S1). These data indicate that the absolute quantitation method of mouse and human dystrophin protein using triple quadrupole HPLC-MS was successfully established.

### 2.3. Protein Quantitation of Dystrophin in Human Muscle Biopsy Specimens

**Figure 5:**
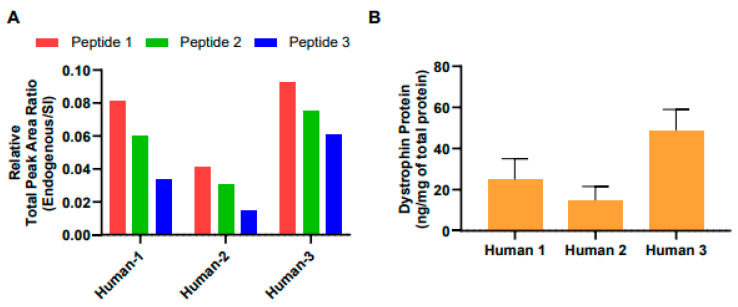
Accurate quantitation of dystrophin protein in human muscle biopsy specimens. (**A**) The relative total area ratio (endogenous/SI) of three dystrophin peptides. (**B**) The amounts of dystrophin protein from three human muscle biopsy samples were quantitated by LC-MS using the three SI-labeled peptides spike-in approach. The data are expressed as the mean ± SE of the quantitative value determined using three dystrophin peptides.

To quantitate dystrophin protein in human muscle biopsy specimens, we analyzed total muscle protein isolated from three human muscle biopsy specimens using the optimized LC-MS method described above. In analyzing human dystrophin, we used 1.2 mg of total muscle protein from human biopsy specimens to quantitate dystrophin protein using the standard internal spike-in method. The relative total peak area ratio of three dystrophin peptides is shown in Figure 5A. The amounts of endogenous dystrophin were determined from the average of the respective dystrophin amounts calculated by the relative total area ratio (endogenous/SI) of three dystrophin peptides. The quantitated total muscle protein in three human biopsy specimens was 24.94, 48.83, and 14.83 ng per mg of total muscle protein, respectively. These amounts were consistent with the presence of the dystrophin protein in skeletal muscle, representing approximately 0.0025%, 0.0049%, and 0.0015% of total muscle protein, respectively (Figure 5B). Thus, we successfully quantitated the amounts of human dystrophin protein in human muscle biopsy specimens.

## 3. Discussion

The lack of dystrophin protein in DMD due to mutation of the *DMD* gene results in severe muscle weakness and degeneration [1,2]. Drug efficacy of exon skipping for DMD treatment is now validated by the demonstration of dystrophin protein restoration using immunoblotting and immunohistochemistry [6,19,28]; however, these methods—which are dependent on antibody specificity—are semiquantitative and do not accurately quantify the protein level. MS-based protein quantitation using mass spectrometry is expected to provide more sensitive and specific quantitation in comparison to immunoblotting and immunohistochemistry.

We succeeded in developing a method for the quantification of dystrophin protein in the specimens of mouse and human skeletal muscle using a triple quadrupole HPLC-MS device. The spike-in approach demonstrated that three custom SI-labeled dystrophin peptides were used as internal standards to quantitate various conditions of the mouse samples (Figure 1 and Figure 2). Using these peptides, the quantitated amounts of dystrophin protein in trial mouse samples were 83.43 ng ± 20.01 (mean ± SE) per mg of total muscle protein in wild-type mice and 107.60 ng ± 22.07 (mean ± SE) per mg of total muscle protein in hDMD-tg/mDMD-null mice, whereas the protein was 1.47 ng ± 1.01 (mean ± SE) in mDMD-null mice (Figure 3 and Figure 4). We further examined the quantitation of the dystrophin protein in human muscle biopsy specimens at 29.53 ± 10.08 (mean ± SE) ng per mg of total muscle protein (Figure 5). In our system, the detection and quantification of the dystrophin protein in mouse and human skeletal muscles were achieved in the initial examinations, indicating that we established an accurate quantitation system for dystrophin protein using triple quadrupole LC-MS.

Recently, several biological approaches have been applied in the attempted quantification of dystrophin protein [21,23,24]. Brown et al. [23] first reported the quantitation of dystrophin protein in human muscle biopsy specimens; the approach quantitated dystrophin protein in 50 μg of total muscle protein from a human biopsy specimen of 0.5 mg, and the quantification sensitivity reached approximately 5% of the normal amount of dystrophin in healthy muscle. Canessa et al. [24] developed a method for the quantitation of dystrophin protein using SILAC-labeled human myotubes and UPLC-MS. In the present study, we quantitated dystrophin protein using the spike-in approach with SI-labeled custom dystrophin peptides, which resulted in the successful detection of dystrophin protein in human biopsy specimens as well as TA from wild-type and hDMD-tg/mDMD-null mice, but not mDMD gene-deficient mDMD-null mice. Our quantitative method using HPLC-MS was able to reach approximately 0.004–0.015% of total muscle proteins in mouse skeletal muscles and approximately 0.001–0.003% of total muscle proteins in three healthy human biopsy specimens. These results indicate that the detection and quantification of a small amount of dystrophin protein using HPLC-MS was successfully achieved.

Dystrophin replacement therapy, including exon skipping, restores the protein level of dystrophin [6]. The expression level of dystrophin protein in DMD patients is <3% of the normal level, while that of patients with severe BMD is 3–10%, and that of patients with mild BMD is ≥20%. Thus, to validate the efficacy of exon skipping, accurate quantitation is needed to at least 3% of the amount of dystrophin in healthy muscle, which is consistent with approximately 0.001% of total muscle protein. In the present study, we detected an average of 83.43 ng, 1.47 ng, and 107.60 ng of dystrophin protein per mg of total muscle protein in wild-type mice, mDMD-null mice, and hDMD-tg/mDMD-null mice, respectively. We successfully quantitated the dystrophin levels in dystrophin-expressing wild-type and hDMD-tg/mDMD-null mice, and also quantitated at least 25% dystrophin protein of hDMD-tg/mDMD-null muscle protein; however, we could not determine whether our method reached sufficient sensitivity to quantitate at least approximately 2.5 ng of dystrophin protein per mg of total muscle protein, consistent with 3% of dystrophin levels in wild-type mice. The next step is to examine the LOD and LOQ, and further quantitate dystrophin protein in human biopsy specimens from BMD and DMD patients.

The present study examined a new approach using synthetic stable isotope-labeled peptides. On the other hand, the data obtained by the approach using SILAC-labeled human primary myotubes contained some errors [23,24]. Therefore, to improve the sensitivity of our quantitation method, we should attempt to apply the SILAC-based approach by using SILAC mice and/or SILAC human myotubes in combination with our spike-in-based approach. We also need to examine the normalization of the amount of dystrophin using another muscle protein as a reference (e.g., alpha-actinin, filamin-C, or myomesin) to further improve the accuracy. This is because fibrofatty replacement is observed in skeletal muscle tissue biopsy specimens from DMD patients [23,24]. Further study is needed to establish the quantification of reference proteins using our method.

For other future techniques, a method using an immunoaffinity chromatography column with a mass spectrometer is currently being developed by several third parties. For instance, anti-peptide Immunoaffinity-LC-MS/MS, also termed stable isotope standards and captured by anti-peptide antibodies (SISCAPA), has been developed for the sensitive and precise detection of protein biomarkers [29,30,31]. The SISCAPA method is a non-gel-based approach that can exclude technical errors in SDS-PAGE and in-gel digestion, and which collects concentrated endogenous peptides using anti-peptide antibodies (e.g., antibody against dystrophin peptide IFLTEQPLEGLEK). These techniques have the potential to further improve the quantitative sensitivity for dystrophin protein.

In conclusion, we successfully developed a quantitation method for dystrophin protein in skeletal muscle extracted from mice and humans using a triple quadrupole HPLC-MS system. The newly established method is applied to various types of LC-MS devices and systems to allow the accurate measurement of the amount of dystrophin protein in patients with DMD in clinics worldwide.

## 4. Materials and Methods

### 4.1. Skeletal Muscles

Male C57BL/6J mice were obtained from Japan SLC Inc. (Shizuoka, Japan). Skeletal muscle from wild-type, mDMD-null, and hDMD-tg/mDMD-null mice were supplied by the National Center of Neurology and Psychiatry (NCNP, Tokyo, Japan). Human skeletal muscle specimens were obtained from ProteoGenex Inc. (Inglewood, CA, USA).

### 4.2. Stable Isotope-Labeled Dystrophin Peptides

Three SI-labeled dystrophin peptides with ^13^C_6_-^15^N_2_-Lysine and Arginine were purchased from SCRUM Inc. (Tokyo, Japan). The sequence and location of the full-length dystrophin protein of each peptide are described in Figure 1A, and the molecular weight (M.W.) is described in Table 1.

### 4.3. LC-MS Analysis

#### Total Protein Extraction from Mouse Skeletal Muscles

Mouse tibialis anterior muscle (TA) and human muscle biopsy samples were isolated and frozen in liquid nitrogen. TA was homogenized in RIPA lysis buffer (150 mM NaCl/25 mM Tris-HCl [pH7.6]/1% NP40/1% sodium deoxycholate/0.1% SDS), and centrifuged at 12,000× *g* for 10 min. The total protein concentration in the collected supernatant was measured by a Bicinchoninic Acid (BCA) protein assay (Pierce BCA Protein Assay Kit; Thermo Fisher Scientific Inc., Waltham, MA, USA).

### 4.4. SDS-PAGE Using 3–8% Tris-Acetate Gel

Total protein from muscle extract was diluted in LDS buffer (NuPAGE LDL Sample Buffer; Thermo Fisher Scientific Inc.) and loaded at 100 μg/lane onto 3–8% Tris-Acetate gel (NuPAGE 3–8% Tris-Acetate Mini Protein Gel; Thermo Fisher Scientific Inc.) for SDS-PAGE at 150 V for 1 h. Protein standard (HiMark Pre-stained Protein Standard; Thermo Fisher Scientific Inc.) was also loaded onto 3–8% Tris-Acetate gel as a molecular weight marker. Gels were stained overnight with GelCode Blue Stain Reagent (Thermo Fisher Scientific Inc.).

### 4.5. In-Gel Tryptic Digestion

A 300–450 kDa section of the gel containing dystrophin protein was excised. Tryptic digestion of dystrophin protein was performed using an In-Gel Tryptic Digestion Kit (Thermo Fisher Scientific Inc.). The method is described briefly as follows. Gel pieces were de-stained by 25 mM ammonium bicarbonate (AB) in 50% acetonitrile. Pieces of gels (8–12) were reduced by TCEP and alkylated by iodoacetamide (IAA). Trypsin (10 ng/μL) was added to the gel pieces. Then, they were incubated at 30 °C overnight with shaking. The digestion mixture was collected, and 1% formic acid solution was added to the gel pieces and incubated for 5 min to further extract peptides and stop trypsin activity. The extraction mixture was added to the digestion mixture, and the sample was ready for the LC-MS analysis.

### 4.6. Protein Quantitation of Dystrophin Protein Using an LC-MS

#### LC-MS Analysis for MRM-Based Protein Quantitation

The liquid chromatographic devices consisted of a Prominence HPLC system (Shimadzu Corp., Kyoto, Japan), including a solvent delivery unit (LC-20AD), autosampler (SIL-20AC_HT_) and column oven (CTO-20AC). The mobile phases consisted of 0.1% formic acid water [A] and 0.1% formic acid acetonitrile [B]. The gradient program for 15.5 min was 15 to 60% [B] for 5 min, 60 to 95% [B] for 0.5 min, 95% [B] for 1 min, then decreasing from 95 to 15% [B]. The flow rate was at 0.2 mL/min, the column oven temperature was 50 °C and the maximum pressure of pumps A and B was 35.0 MPa. A 10 μL analytical sample was injected via an autosampler into a YMC-Triart C18 column (150 × 2.1 mmI.D. S-3 μm, 12 nm; YMC Co., Ltd., Kyoto, Japan).

The MS/MS analysis was performed using a Prominence HPLC system connected to an LCMS-8050 triple quadrupole mass spectrometer (Shimadzu Corp.) in positive ion mode using the multiple reaction monitoring (MRM) technique. The interface was electrospray ionization (ESI). The following parameters were used for the MS/MS analysis: nebulizing gas flow at 3 L/min; heating gas flow at 10 L/min; interface temperature 350 °C; desolvation line temperature 250 °C; heat block temperature 400 °C; drying gas flow at 10 L/min. The Q1 pre-bias, collision energy (CE), and Q3 pre-bias were determined by optimization of LC-MS conditions for the analysis using the synthetic dystrophin peptides in the LabSolutions software program (version 5.113, Shimadzu Corp.) and a quantitative proteomics software program (Skyline, version 21.1, MacCoss Lab Software, University of Washington, Seattle, WA, USA). The pause time was set to 1 msec and the dwell time was set to 24 msec for each MRM transition.

### 4.7. Optimization of LC-MS Conditions for the Analysis of Synthetic Dystrophin Peptides

Three SI-labeled peptides (IFLTEQPLEGLEK, TLNATGEEIIQQSSK, and VLSQIDVAQK) with ^13^C_6_-^15^N_2_-Lysine and Arginine, and unlabeled peptides were synthesized. To determine the optimal condition of LC-MS for the detection of dystrophin peptides, each SI-labeled peptide (10 nM) was mixed with an unlabeled peptide (10 nM), and the mixtures were analyzed. LC-MS conditions were determined to obtain a higher signal intensity of precursor and product ion pair (MRM transition) in cooperation with LabSolutions and Skyline.

### 4.8. Quantitation of Dystrophin in Mouse and Human Skeletal Muscle

Three SI-labeled peptides (10 nM each) were added to samples from mouse and human skeletal muscles prepared by in-gel digestion for the internal standard method. These samples were analyzed by LC-MS using the optimized method. The total peak area ratio (endogenous/SI) in each dystrophin peptide (IFLTEQPLEGLEK, TLNATGEEIIQQSSK, or VLSQIDVAQK) was calculated by the peak area ratio of y-ion fragments per peptide (Table 2). The amount of endogenous dystrophin was calculated from the relative area ratio and the amount of each SI-labeled peptide. The amount of endogenous dystrophin in skeletal muscle was accurately determined from the average amounts of dystrophin calculated using three dystrophin peptides.

### 4.9. Statistical Analysis

The data are expressed as the mean ± standard error of the mean (SE). A one-way ANOVA, followed by Tukey’s test as a post hoc analysis was used for comparisons among three groups. Statistical analyses were performed using GraphPad Prism 10 (Ver. 10.1.0 (316); GraphPad Software, San Diego, CA, USA).

## Figures and Tables

**Table 1:** Sequences of the synthetic dystrophin peptides.

Name	Sequence	SI-Labeled M.W.	Unlabeled M.W.
Peptide 1	IFLTEQPLEGLEK	1524.7	1516.7
Peptide 2	TLNATGEEIIQQSSK	1626.7	1618.7
Peptide 3	VLSQIDVAQK	1108.2	1100.2

**Table 2:** Selected MRM transition exhibiting higher ionization in each peptide.

	Peptide 1	Peptide 2	Peptide 3
**Precursor Ion**	**[IFLTEQPLEGLEK] ^++^**	**[TLNATGEEIIQQSSK] ^+++^**	**[VLSQIDVAQK] ^++^**
Fragment ion 1	y9: [EQPLEGLEK] ^+^	y6: [IQQSSK] ^+^	y6: [IDVAQK] ^+^
Fragment ion 2	y8: [QPLEGLEK] ^+^	y5: [QQSSK] ^+^	y5: [DVAQK] ^+^
Fragment ion 3	y7: [PLEGLEK] ^+^	y4: [QSSK] ^+^	y4: [VAQK] ^+^
Fragment ion 4	y4: [GLEK] ^+^	y3: [SSK] ^+^	y3: [AQK] ^+^

^+^ indicates charge. Gray cells show the precursor ion for each peptide. The y-ions are product ions and C-terminal charged ions from each peptide.

## Data Availability

Data are contained within the article and supplementary material.

## Supplementary Materials

The supporting information can be downloaded at: https://www.mdpi.com/article/10.3390/ijms25010303/s1.

## References

[1] Hoffman E.P., Brown R.H., Kunkel L.M. (1987). Dystrophin: The Protein Product of the Duchenne Muscular Dystrophy Locus. Cell.

[2] Hoffman E.P., Fischbeck K.H., Brown R.H., Johnson M., Medori R., Loire J.D., Harris J.B., Waterston R., Brooke M., Specht L. (1988). Characterization of Dystrophin in Muscle-Biopsy Specimens from Patients with Duchenne’s or Becker’s Muscular Dystrophy. N. Engl. J. Med..

[3] Pasternak C., Wong S., Elson E.L. (1995). Mechanical Function of Dystrophin in Muscle Cells. J. Cell Biol..

[4] Klingler W., Jurkat-Rott K., Lehmann-Horn F., Schleip R. (2012). The Role of Fibrosis in Duchenne Muscular Dystrophy. Acta Myol..

[5] Petrof B.J., Shrager J.B., Stedman H.H., Kelly A.M., Sweeney H.L. (1993). Dystrophin Protects the Sarcolemma from Stresses Developed during Muscle Contraction. Proc. Natl. Acad. Sci. USA.

[6] Tominari T., Aoki Y. (2023). Clinical Development of Novel Therapies for Duchenne Muscular Dystrophy—Current and Future. Neurol. Clin. Neurosci..

[7] Zhao M., Tazumi A., Takayama S., Takenaka-Ninagawa N., Nalbandian M., Nagai M., Nakamura Y., Nakasa M., Watanabe A., Ikeya M. (2020). Induced Fetal Human Muscle Stem Cells with High Therapeutic Potential in a Mouse Muscular Dystrophy Model. Stem Cell Rep..

[8] McDonald C.M., Campbell C., Torricelli R.E., Finkel R.S., Flanigan K.M., Goemans N., Heydemann P., Kaminska A., Kirschner J., Muntoni F. (2017). Ataluren in Patients with Nonsense Mutation Duchenne Muscular Dystrophy (ACT DMD): A Multicentre, Randomised, Double-Blind, Placebo-Controlled, Phase 3 Trial. Lancet.

[9] Min Y.-L., Li H., Rodriguez-Caycedo C., Mireault A.A., Huang J., Shelton J.M., McAnally J.R., Amoasii L., Mammen P.P.A., Bassel-Duby R. (2019). CRISPR-Cas9 Corrects Duchenne Muscular Dystrophy Exon 44 Deletion Mutations in Mice and Human Cells. Sci. Adv..

[10] Moretti A., Fonteyne L., Giesert F., Hoppmann P., Meier A.B., Bozoglu T., Baehr A., Schneider C.M., Sinnecker D., Klett K. (2020). Somatic Gene Editing Ameliorates Skeletal and Cardiac Muscle Failure in Pig and Human Models of Duchenne Muscular Dystrophy. Nat. Med..

[11] Komaki H., Nagata T., Saito T., Masuda S., Takeshita E., Sasaki M., Tachimori H., Nakamura H., Aoki Y., Takeda S. (2018). Systemic Administration of the Antisense Oligonucleotide NS-065/NCNP-01 for Skipping of Exon 53 in Patients with Duchenne Muscular Dystrophy. Sci. Transl. Med..

[12] Drachman D.B., Toyka K.V., Myer E. (1974). Predonisone in Duchenne muscular dystrophy. Lancet.

[13] Angelini C., Peterle E. (2012). Old and New Therapeutic Developments in Steroid Treatment in Duchenne Muscular Dystrophy. Acta Myol..

[14] Komaki H., Maegaki Y., Matsumura T., Shiraishi K., Awano H., Nakamura A., Kinoshita S., Ogata K., Ishigaki K., Saitoh S. (2020). Early Phase 2 Trial of TAS-205 in Patients with Duchenne Muscular Dystrophy. Ann. Clin. Transl. Neurol..

[15] Mohri I., Aritake K., Taniguchi H., Sato Y., Kamauchi S., Nagata N., Maruyama T., Taniike M., Urade Y. (2009). Inhibition of Prostaglandin D Synthase Suppresses Muscular Necrosis. Am. J. Pathol..

[16] Echigoya Y., Nakamura A., Nagata T., Urasawa N., Lim K.R.Q., Trieu N., Panesar D., Kuraoka M., Moulton H.M., Saito T. (2017). Effects of Systemic Multiexon Skipping with Peptide-Conjugated Morpholinos in the Heart of a Dog Model of Duchenne Muscular Dystrophy. Proc. Natl. Acad. Sci. USA.

[17] Cirak S., Arechavala-Gomeza V., Guglieri M., Feng L., Torelli S., Anthony K., Abbs S., Garralda M.E., Bourke J., Wells D.J. (2011). Exon Skipping and Dystrophin Restoration in Patients with Duchenne Muscular Dystrophy after Systemic Phosphorodiamidate Morpholino Oligomer Treatment: An Open-Label, Phase 2, Dose-Escalation Study. Lancet.

[18] Aoki Y., Nakamura A., Yokota T., Saito T., Okazawa H., Nagata T., Takeda S. (2010). In-Frame Dystrophin Following Exon 51-Skipping Improves Muscle Pathology and Function in the Exon 52–Deficient Mdx Mouse. Mol. Ther..

[19] Shimizu R., Ohata M., Tachimori H., Kimura E., Harada Y., Takeshita E., Tamaura A., Takeda S., Komaki H. (2020). Expectations and Anxieties of Duchenne Muscular Dystrophy Patients and Their Families during the First-in-Human Clinical Trial of NS-065/NCNP-01. Brain Dev..

[20] Iftikhar M., Frey J., Shohan M.J., Malek S., Mousa S.A. (2021). Current and Emerging Therapies for Duchenne Muscular Dystrophy and Spinal Muscular Atrophy. Pharmacol. Ther..

[21] Uaesoontrachoon K., Srinivassane S., Warford J., Mekhssian K., Montpetit H., Beauvois R., Keyhani A., Hathout Y., Yamashita T., Satou Y. (2019). Orthogonal Analysis of Dystrophin Protein and MRNA as a Surrogate Outcome for Drug Development. Biomark. Med..

[22] Harding P.L., Fall A.M., Honeyman K., Fletcher S., Wilton S.D. (2007). The Influence of Antisense Oligonucleotide Length on Dystrophin Exon Skipping. Mol. Ther..

[23] Brown K.J., Marathi R., Fiorillo A.A., Ciccimaro E.F., Sharma S., Rowlands D.S., Rayavarapu S., Nagaraju K., Hoffman E.P., Hathout Y. (2012). Accurate Quantitation of Dystrophin Protein in Human Skeletal Muscle Using Mass Spectrometry. J. Bioanal. Biomed..

[24] Canessa E.H., Goswami M.V., Alayi T.D., Hoffman E.P., Hathout Y. (2020). Absolute Quantification of Dystrophin Protein in Human Muscle Biopsies Using Parallel Reaction Monitoring (PRM). J. Mass Spectrom..

[25] Kudoh H., Ikeda H., Kakitani M., Ueda A., Hayasaka M., Tomizuka K., Hanaoka K. (2005). A New Model Mouse for Duchenne Muscular Dystrophy Produced by 2.4 Mb Deletion of Dystrophin Gene Using Cre-LoxP Recombination System. Biochem. Biophys. Res. Commun..

[26] AC’t Hoen P., de Meijer E.J., Boer J.M., Vossen R.H., Turk R., Maatman R.G., Davies K.E., van Ommen G.J., van Deutekom J.C., den Dunnen J.T. (2008). Generation and Characterization of Transgenic Mice with the Full-Length Human DMD Gene. J. Biol. Chem..

[27] Echigoya Y., Lim K.R.Q., Trieu N., Bao B., Nichols B.M., Vila M.C., Novak J.S., Hara Y., Lee J., Touznik A. (2017). Quantitative Antisense Screening and Optimization for Exon 51 Skipping in Duchenne Muscular Dystrophy. Mol. Ther..

[28] Roshmi R.R., Yokota T. (2019). Viltolarsen for the Treatment of Duchenne Muscular Dystrophy. Drugs Today.

[29] Neubert H., Fan Y.-Y., Ocaa M.F. (2016). Quantification of Protein Biomarkers in Tissues: New Capabilities with Pellet Digestion Peptide Immunoaffinity LCMS/MS. Bioanalysis.

[30] Razavi M., Anderson N.L., Pope M.E., Yip R., Pearson T.W. (2016). High Precision Quantification of Human Plasma Proteins Using the Automated SISCAPA Immuno-MS Workflow. New Biotechnol..

[31] Farrokhi V., Walsh J., Palandra J., Brodfuehrer J., Caiazzo T., Owens J., Binks M., Neelakantan S., Yong F., Dua P. (2022). Dystrophin and Mini-Dystrophin Quantification by Mass Spectrometry in Skeletal Muscle for Gene Therapy Development in Duchenne Muscular Dystrophy. Gene Ther..

