# Supplementary material for: Establishment of a Triple Quadrupole HPLC-MS Quantitation Method for Dystrophin Protein in Mouse and Human Skeletal Muscle
Source: Int J Mol Sci. 2023 Dec 25;25(1):303. doi: 10.3390/ijms25010303 (PMC10779312; doi:10.3390/ijms25010303)
Supplement: Supplementary file 1 [file ijms-25-00303-s001.zip › ijms-2741891-supplementary.pdf]

Supplementary Material

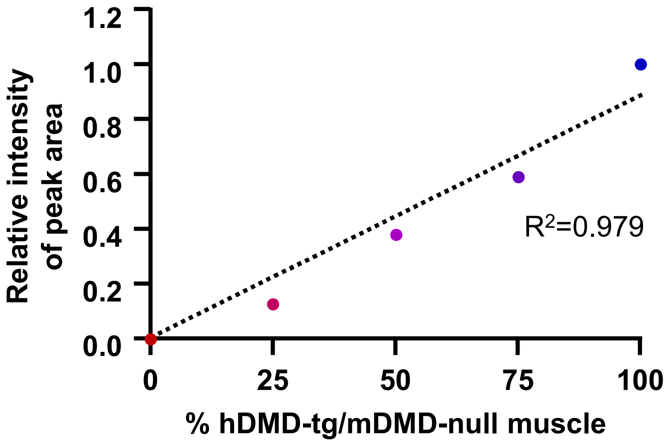

Figure S1: Standard curve of dystrophin protein.
LC-MS analysis was performed using samples of hDMD-tg/mDMD-null muscle lysate mixed with mDMD-null muscle lysate at the indicated ratios. 0%, 25%, 50%, 75%, and 100% indicated the mixtures of 0%:100%, 25%:75%, 50%:50%, 75%:25%, and 100%:0% (hDMD-tg/mDMD-null muscle lysate to mDMD-null muscle lysate), respectively.
